# Genome size variation within *Crithmum maritimum*: Clues on the colonization of insular environments

**DOI:** 10.1002/ece3.10009

**Published:** 2023-04-19

**Authors:** Guilherme Roxo, Miguel Brilhante, Mónica Moura, Miguel Menezes de Sequeira, Luís Silva, José Carlos Costa, Raquel Vasconcelos, Pedro Talhinhas, Maria M. Romeiras

**Affiliations:** ^1^ Linking Landscape, Environment, Agriculture and Food (LEAF), Associated Laboratory TERRA, Instituto Superior de Agronomia (ISA) Universidade de Lisboa, Tapada da Ajuda Lisbon Portugal; ^2^ CIBIO‐Azores, Departamento de Biologia Universidade dos Açores Ponta Delgada Portugal; ^3^ BIOPOLIS Program in Genomics, Biodiversity and Land Planning CIBIO Centro de Investigação em Biodiversidade e Recursos Genéticos, Campus de Vairão Vairão Portugal; ^4^ Madeira Botanical Group, Faculty of Life Sciences University of Madeira Funchal Portugal; ^5^ Centre for Ecology, Evolution and Environmental Changes (cE3c) & CHANGE—Global Change and Sustainability Institute, Faculdade de Ciências Universidade de Lisboa Lisbon Portugal

**Keywords:** DNA flow cytometry, genome‐climate interactions, intraspecific variation, Macaronesian Islands, population studies

## Abstract

Angiosperms present an astonishing diversity of genome sizes that can vary intra‐ or interspecifically. The remarkable new cytogenomic data shed some light on our understanding of evolution, but few studies were performed with insular and mainland populations to test possible correlations with dispersal, speciation, and adaptations to insular environments. Here, patterns of cytogenomic diversity were assessed among geographic samples (ca. 114) of *Crithmum maritimum* (Apiaceae), collected across the Azores and Madeira archipelagos, as well as in adjacent continental areas of Portugal. Using flow cytometry, the results indicated a significant intraspecific genome size variation, spanning from reduced sizes in the insular populations to larger ones in the mainland populations. Moreover, there was a tendency for an increase in genome size along the mainland populations, associated with lower temperatures, higher precipitation, and lower precipitation seasonality. However, this gradient might be the result of historic phylogeographical events associated with previous dispersal and extinction of local populations. Overall, our findings provided evidence that smaller genome sizes might play a critical role in the colonization of islands, corroborating other studies that argue that organisms with smaller genomes use fewer resources, having a selective advantage under insular environments. Although further studies are needed to improve our understanding of the mechanisms underlying genome size evolution on islands, conservation strategies must be promoted to protect the rich cytogenomic diversity found among *C. maritimum* populations, which occur in coastal areas that are particularly threatened by human activity, pollution, invasive species, and climate changes.

## Graphical abstract

Angiosperms present an astonishing diversity of genome sizes that can vary intra‐ or interspecifically. Patterns of cytogenomic diversity were assessed among geographic samples (ca. 114) of *Crithmum maritimum* (Apiaceae), collected across Azores and Madeira archipelagos, as well as in adjacent continental areas. Our findings provided evidence that smaller genome sizes might play a critical role in the colonization of islands, corroborating other studies that argue that organisms with smaller genomes use fewer resources, having a selective advantage under insular environments.

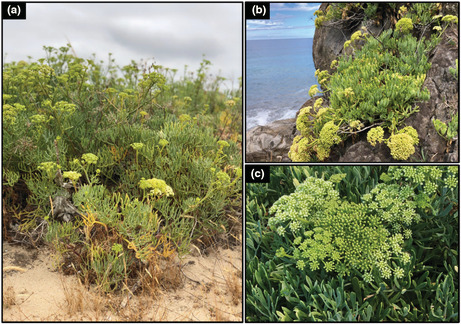

## 1 INTRODUCTION

Angiosperms present an amazing diversity of genome sizes (GS) (Pellicer et al., 2018), spanning the 1C‐values from 0.065 picograms (pg) in *Genlisea aurea* A.St.‐Hill. (Fleischmann et al., 2014) to 152.23 pg in *Paris japonica* Franch (Pellicer et al., 2010). Intraspecific genome size variation has also been reported for several taxa (Gregory, 2001, 2005); for example, a variation of 16% was reported for *Brassica rapa* (Boutte et al., 2020), 9.2% for *Lupinus mutabilis* Sweet (Guilengue et al., 2020), and 21%–26% for *Sinningia speciosa* (G.Lodd. ex Ker Gawl.) Hiern (Zaitlin & Pierce, 2010). Moreover, it has been proposed that the intraspecific C‐value variation can resolve complex low‐level taxonomies, including the delimitation of species boundaries, even if not to be used alone as a taxonomic marker (Afonso et al., 2021). For instance, Murray (2005) proposed that two taxa should be recognized as distinct species for *Lachnagrostis littoralis* (Hack.) Edgar (i.e., subsp. *littoralis* and subsp. *salaria*) based on cytogenomic, morphological and ecological data.

Polyploidization, and more recently, transposable elements have been recognized as important sources of speciation (Craddock, 2016; Wood et al., 2009). Both phenomena lead initially to an increase in the genome size (Pellicer et al., 2018); nevertheless, it is important to note that these processes are reversible, being the plants equipped with mechanisms that lead to genome downsizing (e.g., chromosomal rearrangement, large‐scale loss of repetitive sequences and duplicated genes, elimination of transposed copies) (Chen et al., 2007; Freeling et al., 2012; Leitch & Leitch, 2008; Vitte & Panaud, 2005; Wendel, 2015).

Moreover, GS can also have a significant impact at the ecological and evolutionary level (Biémont, 2008), since it correlates not only with several phenotypic traits, such as flowering time, flower size, seed mass, and photosynthetic rate (e.g., Beaulieu, Leitch, & Knight, 2007, Beaulieu, Moles, et al., 2007, 2008; Meagher & Vassiliadis, 2005) but also with environmental variables, namely altitude, latitude, and temperature (e.g., Knight et al., 2005; Suda et al., 2005), and it was shown to have implications in ecological adaptation in plants (e.g., Ramsey, 2011). In fact, it was discovered that the GS is negatively linked with altitude in the *Zea* (Díez et al., 2013) and *Berberis* (Bottini et al., 2000) genera.

Although, remarkable cytogenomic data are shedding some light on our understanding of evolution, few studies were performed with plant lineages from Macaronesian Islands (i.e., Azores, Madeira, Selvagens, Canary Islands, and Cabo Verde) which harbors a rich endemic flora, of approximately 900 vascular plant species (Florencio et al., 2021). Since the seminal study of Suda et al. (2003), using the Canary endemic flora, few studies were performed in Macaronesia. Furthermore, several correlations with environmental variable were found in Suda et al. (2003, 2005), for example, the *Argyranthemum*, *Micromeria*, and *Silene* genera presented positive correlations between mean annual temperature and GS and negative correlations between GS and rainfall and altitude. On the other hand, the opposite trend was observed in the *Crambe* and *Sonchus* genera. Brilhante et al. (2021) found negative correlations between annual mean precipitation and GS for *Aeonium* in Tenerife.

**FIGURE 1:**
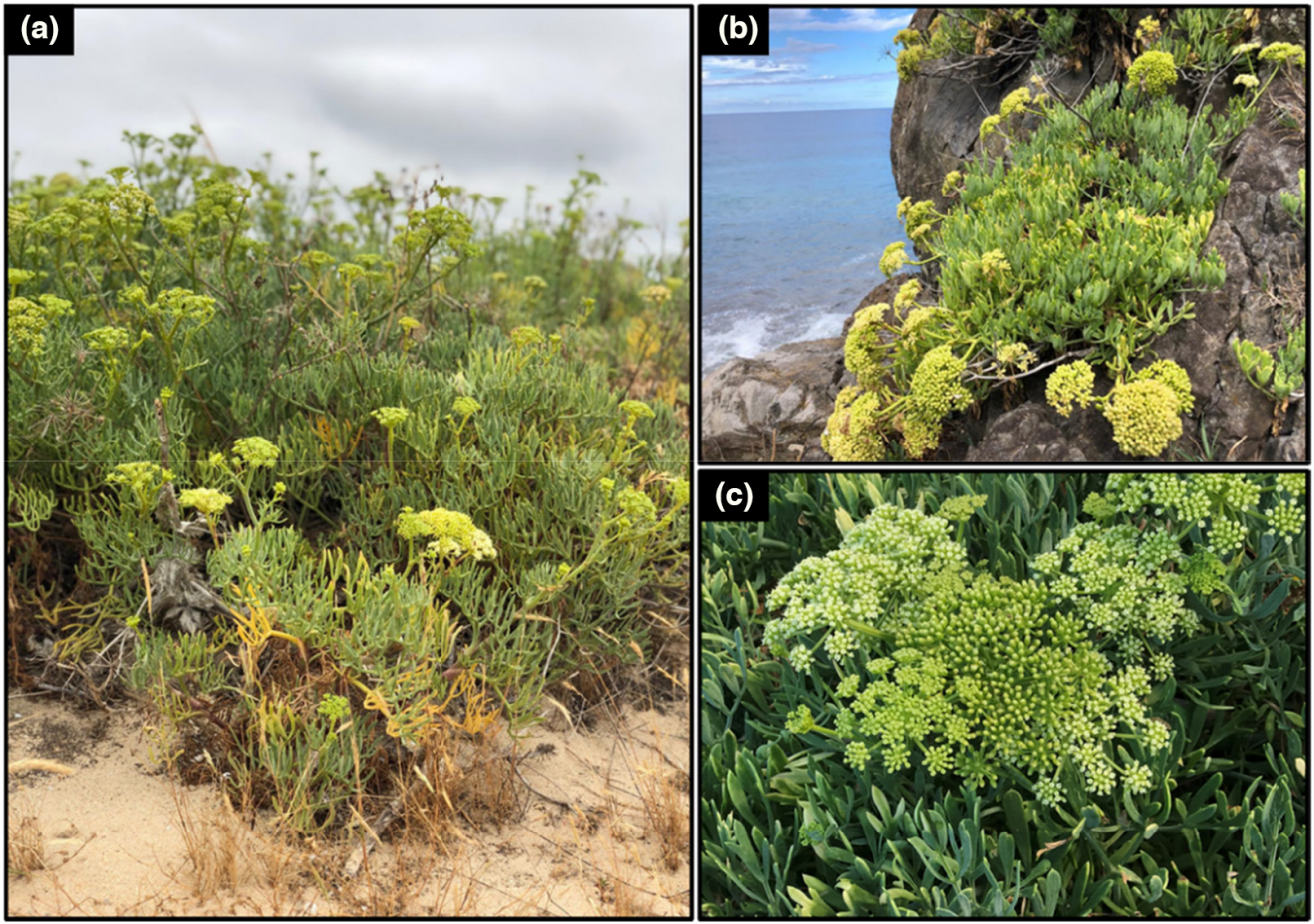
Plants of *Crithmum maritimum* (a) growing in sands in Viana do Castelo; (b) growing in coastal rocky cliffs in São Jorge, Madeira; and (c) detail of its inflorescences. (Photos by Guilherme Roxo and Maria Romeiras).

Since GS correlates with phenotypic and ecological traits it can be inferred that GS is shaped by natural selection, nonetheless, genetic drift has also been proposed to explain GS variation (Oliver et al., 2007; Whitney et al., 2010). To test if GS undergoes selection, population‐level analyses are ideal (Díez et al., 2013). The sea fennel or rock samphire *Crithmum maritimum* L. (Apiaceae), a monospecific genus (Castroviejo, 2003; Meot‐Duros & Magné, 2009), is a facultative halophyte that grows in rocky sea cliffs and occasionally in sands and gravel (Figure 1). It has a very wide distribution, occurring along the European Atlantic coasts, the Azores, Madeira, and Canary archipelago, the Mediterranean and Black Sea coast, and northwest Africa, with its distribution being limited by temperature (Castroviejo, 2003; Crawford, 1982). However, due to climate change, *C. maritimum* distribution is currently expanding northwards (Metzing & Gerlach, 2001). *C. maritimum* is an aromatic herb with therapeutic healing properties known since ancient times and it was mentioned by Hippocrates in the 4th century BCE to soothe vesical pains (Pline, 1957), and used by sailors who ate *C. maritimum* fresh leaves to prevent scurvy (Baytop, 1984). Over the centuries, the use of this plant has decreased, but in the 21st century more studies have been published suggesting its potential as a crop (e.g., Renna, 2018). In fact, its leaves hold high contents of carotenoids, flavonoids, vitamin C, and other bioactive substances (Özcan et al., 2001). Moreover, recent studies identified high levels of both genetic (Latron et al., 2018, 2020) and phytochemical differentiation among *C. maritimum* populations (Katsouri et al., 2001; Kulišic–bilušic et al., 2010; Maleš et al., 2001). However, cytogenomically, *C. maritimum* was analyzed only from one location (i.e., Strunjan saltpan in Slovenia), resulting in an estimation of a 2C‐value of 4.38 pg (Koce et al., 2008).

The present study aimed to investigate the cytogenomic variation of *C. maritimum* at the population level, and if it correlated with environmental variables. Namely, we intended to determine the amount of variation of GS among populations, and to test an association with a geographical or environmental variables. Additionally, we discussed our results from an evolutionary and taxonomic point of view, in accordance with the current theories of genome evolution.

## 2 MATERIALS AND METHODS

### 2.1 Climatic characterization and biogeographic regions of the study area

The Azorean archipelago belongs to the Atlantic European Province (Costa et al., 1998) and is located in the North Atlantic ridge between the latitudes of 36° 45′N and 39° 43′N and the longitudes of 24° 45′W and 31° 17′W. Its climate is wet, cloudy, and with mild temperatures all year round. This is because from September to March the Azores is crossed by the North Atlantic Storm‐track and the rest of the year by the Azores anticyclone.

The Madeira archipelago belongs to the Madeirese Province (Costa et al., 1998) and is situated to the Southeast of continental Portugal between 32° 24′ and 33° 27′N in latitude and 16° 16′ and 17° 16′W in longitude. The climate is influenced by trade winds from the N and NE and by its orography. The Northern slopes are more humid and have higher rainfall and less sunlight compared to the southern slope. The mean annual air temperature is between 8°C in the highest peak and 18–19°C in the coastal region.

The mainland Portugal climate is characterized by a temperate climate with rainy winters and hot, dry summers. Across its territory nine biogeographic regions can be recognized (Costa et al., 1998) from which four were included in our sampling. The northernmost sector is the Galician Portuguese Sector which belongs to the Eurosiberian Region being characterized by a temperate and rainy climate without a clear dry season. The other three sectors belong to the Andalusian Lusitanian Coastal Province which is characterized by mild climate. The Portuguese Divisorian Sector the most northern one of the three is mostly located in the lower mesomediterranean level; however, the coastal zones are in the sub‐humid upper levels of the thermomediterranean climate. The Ribataganian‐Sadese Sector is characterized by a thermomediterranean sub‐humid climate and the Algarvese‐Monchiquense Sector by a thermomediterranean dry to subhumid climate.

### 2.2 Sampling

**FIGURE 2:**
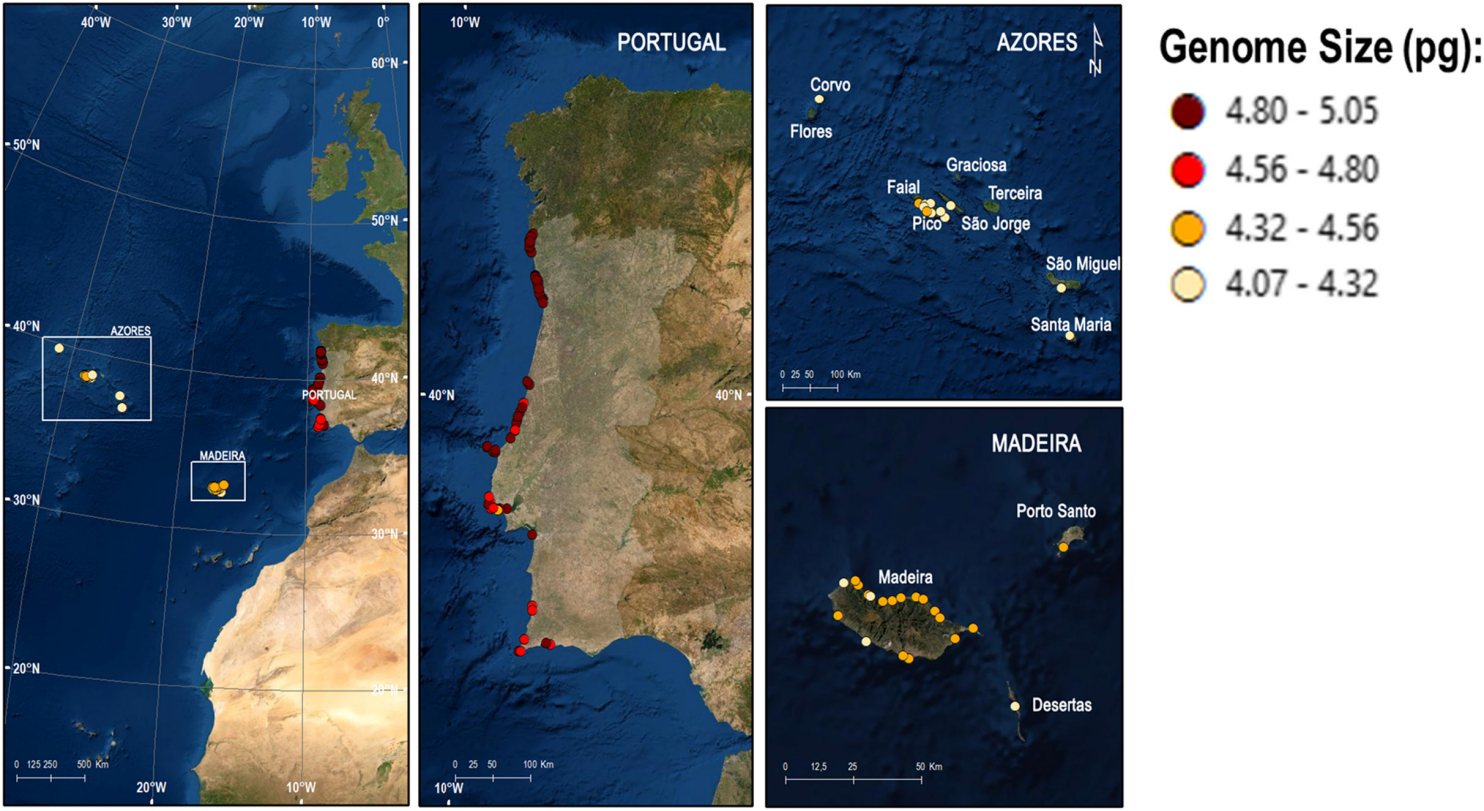
Distribution map with the dots showing the gradient of genome size across the studied areas. The genome size (in picograms, pg) per sampled populations is mapped according to the color of the bubbles.

Specimens of *C. maritimum* were collected for cytogenomic studies during several field surveys (2017–2020), in mainland Portugal and the archipelagos of the Azores and Madeira. Particularly, fieldwork took place on six Azorean islands (Corvo, Faial, Pico, Santa Maria, São Jorge, São Miguel), three Madeiran islands (Deserta Grande, Madeira, Porto Santo). A total of 114 populations were sampled (Figure 2; for data see Appendix S1—Table S1). From each population, a minimum of three specimens was collected. For each site, we recorded geographical coordinates and altitude with a GPS device. Each sample was collected and preserved in wet tissue paper, wrapped in aluminum foil and ziplock bags, then preserved at 5°C, and posted to the laboratory.

Vouchers from most of the samples (at least one per population) were deposited at Herbarium of Instituto Superior de Agronomia, University of Lisbon (LISI), at the Herbarium of the University of Madeira (UMAD) and at the Herbarium of the University of the Azores (AZB).

### 2.3 Cytogenomic analysis

Nuclear DNA content was estimated using FCM. Preparation of suspensions of intact nuclei for analysis was performed following the method of Galbraith et al. (1983). The fresh young leaves were chopped with a razor blade in a Petri dish containing 1 mL of Woody Plant Buffer (WPB 0.2 M Tris–HCl, 4 mM MgCl_2_, 1% Triton X‐100, Na_2_EDTA 2 mM, NaCl 86 mM, sodium metabisulfite 20 mM, 1% PVP‐10, pH 7.5; Loureiro et al., 2007). The nuclear suspension was sieved using a nylon mesh with 30 μm to remove large debris. Then, nuclei were stained with 25 μg ml^−1^ and a volume of 50 μL of propidium iodide (PI; Sigma‐Aldrich, USA). To estimate the nuclear DNA content, DNA from *Solanum lycopersicum* L. ‘Stupické’ (2C = 1.96 pg; Doležel et al., 1992) was used as reference standard. The acquisition of numeric data and fluorescence graphs was made by Sysmex FloMax software v2.4d (Sysmex, Görlitz, Germany), as described by Guilengue et al. (2020). The histograms for each sample were recorded and the C‐values were calculated with the following formula:

$$\text{Nuclear}\;\mathrm{DNA}\;\text{Content}\;\left(\mathrm{pg}\right)=\frac{\text{Sample}\;G1\;\text{Peak Mean}\times \mathrm{GS}\;\text{of Reference Standard}}{\text{Reference Standard}\;G1\;\text{Peak Mean}}$$

### 2.4 Statistical analysis

Statistical analyses and descriptive statistics were performed using R v4.2.21 software (R Core Team, 2020). We followed the same general approach already outlined by our team in a previous paper (Roxo et al., 2021), which is summarized below.

#### 2.4.1 Basic statistics

Mean values and standard deviations (SD) of the genome size (2C‐values) were calculated for each population, and descriptive analyses were based on boxplots. Comparisons of GS values were made: (i) among populations; (ii) among island and mainland populations; and (iii) among mainland biogeographic regions and the two archipelagos. Box–Cox or other conventional transformation techniques (Box & Cox, 1964; Zar, 2010) did not normalize our data (*p* < .05 with the Shapiro–Wilk test) (Shapiro & Wilk, 1965). Thus, group comparisons were carried out with nonparametric tests. The Mann–Whitney and Kruskal–Wallis tests were performed for comparisons between two groups or more than two groups, respectively, and in the case of a rejection of the null hypothesis, the latter was followed by nonparametric multiple comparison test (Conover & Iman, 1979; Siegel & Castellan, 1988), using the function posthoc.kruskal.conover.test of the “The Pairwise Multiple Comparison of Mean Ranks Package (PMCMR)” R package (Pohlert, 2014), This function allows a Bonferroni‐type adjustment of *p*‐values to ensure a high level of statistical power, by reducing the probability of performing a type II error.

#### 2.4.2 Generalized linear models

To assess the factors affecting the 2C‐values, we calculated Gaussian generalized linear models (GLMs) following two scenarios: (i) the first including island and mainland populations and (ii) the second including mainland populations only. A null model was used as benchmark in both cases. In the first scenario, we calculated a biogeographic model (among mainland biogeographic regions and the two archipelagos), an island model (comparing islands with mainland populations) a bioclimatic model (including the principal components extracted from a principal component analysis, PCA, applied to the 19 bioclimatic variables, BIOCLIM), a full model including all the previous factors, and an intermediate model resulting from its simplification. In the second scenario, we included a biogeographic model, a latitude model, a longitude model, an altitude model, a bioclimatic model (including the principal components extracted from a PCA applied to the 19 bioclimatic variables), a full model including all the previous factors, as well as other models resulting from its simplification. We implemented this analysis with the GLM function of R, following previous work in this area by our team, namely Ávila et al. (2018), Parelho et al. (2021), and Roxo et al. (2021) (see references therein). Model selection was based on the maximum likelihood approach using the Akaike's information criterion (AIC). The model with the lowest AIC and the highest *R* ^2^ was considered to best fit the data. The evaluation of the GLMs was performed using the R package “mass.” Following Roxo et al. (2021), despite our raw data was not normal, we included all the samples in the analysis and, according to the central limit theorem, when independent random variables are added, their properly normalized sum tends to a normal distribution, independently of the original variable distribution. That is, with a large sample size (i.e., more than 100 observations in this case) the mean values tend to a normal distribution (Kwak & Kim, 2017). Therefore, as suggested in a previous work by our team (Roxo et al., 2021), we considered that the application of the GLMs is appropriate.

#### 2.4.3 Bioclimatic factors

Nineteen climatic variables were used in the present study (for data see Appendix S1—Table S2) and extracted from CHELSA dataset version 1.2 (Climatologies at High Resolution for the Earth's Land Surface Areas, available at https://chelsa‐climate.org; Karger et al., 2017, 2020).

To extract the principal components of the 19 bioclimatic variables, we used the “vegan” package for R and followed the Kaiser–Guttman and broken stick model criteria to determine the number of components to retain, that is, those with eigenvalues above the mean eigenvalue and the broken stick model (see Borcard et al., 2011). We then interpreted the bioclimatic meaning of the retained components, based on their correlations (i.e., loadings) with the initial bioclimatic variables, and determined the amount of variation explained by each retained component, based on their respective eigenvalues.

## 3 RESULTS

### 3.1 Genome size variation in islands and mainland populations

**FIGURE 3:**
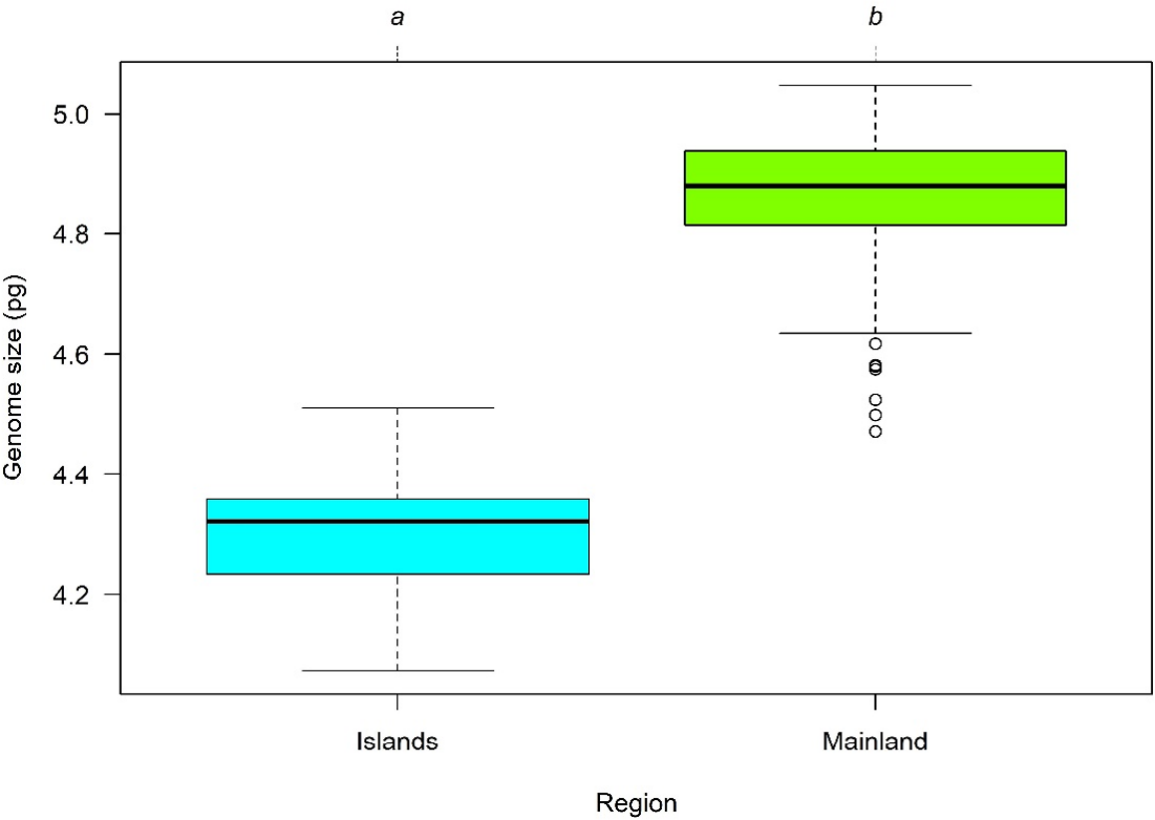
Boxplot diagram showing the genome size variation in *Crithmum maritimum* populations sampled in mainland Portugal, and in the archipelagos of Azores and Madeira. The plot represents the four quartiles; the dots represent the outliers. Different letters indicate a significant difference (*p* < .05) according to the Mann–Whitney test (W = 5923, *p* = 2.2e‐16).

The cytogenomic results for the 114 populations were summarized in Appendix S1—Table S3. The global mean GS corresponded to 4.710 ± 0.294 pg, while the coefficient of variation ranged from 0.840 to 6.928% (mean = 2.445%). A high cytogenomic diversity across the different populations was observed with mainland populations showing larger GS values (4.859 ± 0.148 pg) compared to the island populations (4.332 ± 0.198 pg; Figure 3).

**FIGURE 4:**
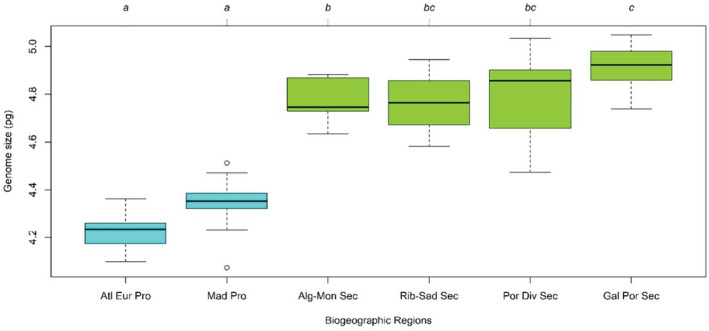
Boxplot diagram showing the genome size variation in *Crithmum maritimum* populations across the biogeographic regions of the Portuguese Territory (i.e., Alt Eur Pro, Atlantic European Province; Mad Pro, Madeirese Province; Alg‐Mon Sec, Algarvese‐Monchiquense Sector; Rib‐Sad Sec, Ribataganian‐Sadese Sector; Port Div Sec, Portuguese Divisorian Sector; Gal Por Sec, Galician Portuguese Sector). The plot represents the four quartiles; the dots represent the outliers. Different letters indicate significant differences (*p* < .05) according to the results of a nonparametric multiple comparison test applied after the Kruskal–Wallis test (Chi‐squared = 81.706, df = 5, *p* = 3.688e‐16).

Regarding the islands, the Azorean population presented the smallest mean 2C‐values (4.217 ± 0.093 pg), followed by Madeira (4.348 ± 0.107 pg). In the mainland, a geographic gradient can be observed, with the northern populations presenting larger genomes in comparison to the southern ones (see Figure 2). Overall, Fajã das Achadas da Cruz in Madeira Island presented the smallest genome (4.074 ± 0.079 pg), and Praia dos Barcos, Porto, in the mainland, the largest one (5.047 ± 0.145 pg; for data see Appendix S1—Table S3). Regarding the biogeographic regions by Costa et al. (1998), once again the islands populations presenting the smallest genomes (i.e., Atlantic European Province and Madeirese Province) and a gradient in the mainland can be seen with the highest 2C‐values in the Northernmost sector, the Galician Portuguese Sector (4.918 ± 0.073 pg), followed by the Portuguese Divisorian Sector (4.787 ± 0.152 pg), Algarvese‐Monchiquense Sector (4.771 ± 0.092 pg) and with the smallest values the Ribataganian‐Sadese Sector (4.763 ± 0.149 pg) (Figure 4).

### 3.2 Climatic variables and genome size

When considering the whole dataset, the best GLM corresponded to the junction of the biogeographic regions with the bioclimatic variables (Table 1), separating Azores and Madeira from the biogeographic regions in the mainland, which agrees with the non‐parametric analysis shown in Figures 3 and 4. Although the differences between islands and the mainland were significant (Figure 3), this model was less informative than the model discriminating biogeographic regions both in islands and mainland (Figure 4). The latter model not only allowed to clearly separate Azores and Madeira from the mainland populations, but also incorporated the considerable variation found among the latter. Regarding the bioclimatic variables, we retained three main components characterizing the climate to be found at all included populations (Table 2; for data see Appendix S1—Figure S1). The first component (PC1) was mostly associated with high temperatures, particularly during the coldest/wettest part of the year, with low values for temperature variation and with higher precipitation in the warmest part of the year. The second component (PC2) was associated with high temperatures, low precipitation seasonality, and high precipitation. The third component (PC3) was associated with low precipitation on the driest part of the year but with high precipitation during the wettest/coldest part of the year and with high precipitation seasonality. The three retained components explained 51.2%, 26.2% and 13.8% of the variation in the data, respectively. The addition of the bioclimatic variables in the GLMs (i.e., Biogeographic + BIOCLIM) also revealed to be an adequate GLM model (Table 1). However, since it did not show any relevant improvement in terms of AIC or *R* ^2^ values when compared to the model that only included the biogeographic regions, it can be inferred that the geographic differences between mainland and insular regions have a greater role in shaping genome size.

**TABLE 1:** Results of the application of Gaussian generalized linear models to the *Crithmum maritimum* genome size data for all the samples or for mainland data only.

Data	Models	AICc	*R* ^2^
All	Biogeographic + BIOCLIM	−183.829	0.8826203
	Biogeographic	−176.790	0.8671450
	Islands + BIOCLIM	−174.022	0.8611394
	BIOCLIM	−155.962	0.8340893
	Islands	−148.116	0.8153759
	Null	42.3698	‐
Mainland	BIOCLIM	−120.616	0.3403528
	Latitude	−120.243	0.3183408
	Longitude + BIOCLIM	−119.898	0.3532768
	Altitude + BIOCLIM	−119.263	0.3480630
	Latitude + BIOCLIM	−118.948	0.3454568
	Biogeographic + BIOCLIM	−118.768	0.3822901
	Biogeographic	−109.701	0.2641737
	Longitude	−107.888	0.2029487
	Null	−92.1304	‐
	Altitude	−91.1151	0.0144120

*Note*: The null model included the intercept only and was used as benchmark. Models with lowest AICc (Akaike's Information Criterion, corrected) and highest *R* ^2^ (adjusted determination coefficient) correspond to the best fit (less information loss). BIOCLIM correspond to the three (all data) or two (mainland data) principal components extracted from the 19 bioclimatic variables.

**TABLE 2:** Correlation of PC axes with bioclimatic variables extracted, including all data and only mainland data, respectively.

Variable		All data			Mainland	
Description	Code	PC1	PC2	PC3	PC1	PC2
Annual Mean Temperature	BIO01	1.2055	−0.9658	0.02266	1.3358	0.16801
Mean Diurnal Range	BIO02	−1.4892	0.1808	0.32628	0.1413	−1.40061
Isothermality	BIO03	−1.4867	0.1874	0.38696	0.2999	−1.30236
Temperature Seasonality	BIO04	−1.4565	0.3530	0.03263	−0.2891	−1.36900
Max. temperature of warmest month	BIO05	−0.9998	−0.7736	0.17144	0.8519	−1.08714
Min. temperature of coldest month	BIO06	1.4318	−0.5967	−0.14422	0.8354	1.10800
Temperature annual range	BIO07	−1.4903	0.2437	0.17180	−0.0134	−1.41329
Mean temperature of wettest quarter	BIO08	1.2873	−0.7814	−0.20812	1.1371	0.75212
Mean temperature of driest quarter	BIO09	0.1153	−1.0839	0.84831	1.0554	−0.73100
Mean temperature of warmest quarter	BIO10	0.7162	−1.1097	−0.06649	1.0554	−0.73100
Mean temperature of coldest quarter	BIO11	1.3715	−0.7382	−0.08218	1.1388	0.77556
Annual precipitation	BIO12	0.9689	1.0162	0.64521	−1.3763	0.06476
Precipitation of wettest month	BIO13	0.9832	0.8671	0.83583	−1.3488	0.12659
Precipitation of driest month	BIO14	0.5668	1.0051	−0.89236	−1.3800	0.13383
Precipitation seasonality	BIO15	−0.1833	−0.7656	1.17066	1.3159	0.38142
Precipitation of wettest quarter	BIO16	0.9683	0.8956	0.82172	−1.3543	0.14575
Precipitation of driest quarter	BIO17	0.7114	1.0949	−0.67493	−1.3882	0.08775
Precipitation of warmest quarter	BIO18	1.4198	0.4623	0.16872	−1.3882	0.08775
Precipitation of coldest quarter	BIO19	0.8290	0.9152	0.92190	−1.3383	−0.10320

When considering mainland data only, the fine information provided by the climate (i.e., the two main climatic components extracted from the bioclimatic data) (see Table 2, for data see Appendix S1—Figure S1), provided the best GLM model (Table 1). PC1 was associated with high temperatures, low precipitation, and high precipitation seasonality. PC2 was associated with low temperature variation, low temperature in the driest/ warmest part of the year, high temperature in the coldest/wettest part of the year. The two retained components explained 60.7% and 32.7% of the variation in the data, respectively. Latitude and longitude provided significant but relatively low fit models, while altitude provided a non‐significant model (Table 1). Our results seem to demonstrate a gradient from south to north (with GS increasing in that direction, Spearman correlation, *r* = 0.46, *p* < .001) and from east to west (with GS decreasing in that direction, Spearman correlation, *r* = 0.42, *p* < .001), the latter being the result of smaller genomes in island populations. The correlation of GS with the second main climatic component was negative (Spearman correlation, *r* = −0.28, *p* = .01481) meaning that larger values of GS would be found at places with high temperature variation, high temperature in the driest/warmest part of the year, and low temperature in the coldest/wettest part of the year (see Table 2). The correlation of GS with the first main climatic component was negative (Spearman correlation, r = −0.44, *p* < .001) meaning that larger values would be found at places with low temperatures, high precipitation, and low precipitation seasonality, reinforcing the possibility of a positive gradient of GS from southern to northern mainland Portugal.

## 4 DISCUSSION

Despite the expansion of flow cytometry studies (Loureiro et al., 2010), most studies have focused on the level of polyploidy across different populations (Padilla‐Garcia et al., 2018) or the interspecific variation in closely related taxa (Castro et al., 2013; Roxo et al., 2021; Zahradníček et al., 2018). There are few studies which investigate the intraspecific variation among insular and continental populations, with only Walker et al. (2006) investigating it across *Bituminaria bituminosa* (L.) C.H. Stirton (Fabaceae). Regarding *C. maritimum*, it has a great cytogenomic diversity, which corroborates with the studies of Latron et al. (2018, 2020) that used nuclear microsatellites.

### 4.1 Genome size variation and colonization of insular environments

Our results revealed that the island populations presented smaller genome sizes and less variation when compared to mainland ones. Moreover, the data collection of 114 populations across the Portuguese territory revealed that the biogeography, separating Azores, Madeira and the biogeographic region in the mainland plays critical role in shaping genome size. When considering the entire dataset, geographic isolation and the distinction between insular and continental habitats appear to be the most important factors in shaping genome size. The tendency toward smaller genome size in endemic species was observed by Suda et al. (2003) and (2005) in the Canary Islands, and by Kapralov and Filatov (2011) in the Hawaiian and Marquesas archipelagos. Therefore, it seems that small genomes are advantageous when colonizing new habitats. Kapralov and Filatov (2011) argue that smaller island genome sizes may be due to: (i) genome size downsizing during or after colonization, or (ii) predominance of colonizers with small genomes. Mechanisms underlying angiosperm genome size variation have only recently been better understood, with several correlations between genome size and ecological and evolutionary factors being investigated (Roddy et al., 2020).

At the molecular level, Suda et al. (2005) argues that smaller genomes of island samples might be more advantageous by reducing genetic instability. Furthermore, plants may not be able to colonize habitats with low phosphate and nitrogen levels if they have large genomes (Guignard et al., 2016; Šmarda et al., 2013). At the cellular level, large genomes imply larger nuclei, which in turn produce larger cells, whereas small genomes are more flexible in terms of cell size, and as cell size decreases, the ratio of cell surface area to cell volume rises exponentially (Roddy et al., 2020). Therefore, genome size indirectly restricts the maximum rate of stomatal opening and closing by having an impact on the sizes and densities of stomata (Drake et al., 2013; McAusland et al., 2016; Roddy et al., 2020), which in turn has an influence on maximum rates of leaf surface conductance to CO_2_, water, and ultimately photosynthetic metabolism per unit leaf surface area (Simonin & Roddy, 2018). Moreover, it appears from research on invasive species that invasive genotypes have smaller genomes and faster rates of stem elongation than their native genotypes (Lavergne et al., 2010), and studies with maize have established a negative association between genome size and the rate of cell production (Bilinski et al., 2018). Therefore, it seems that small genomes allow for greater variation in cell size and metabolism, facilitating the structure's adaptation to environmental changes (Knight et al., 2005; Roddy et al., 2020). Moreover, GS also seems to be correlated with breeding systems (Bennett, 1972) and *Crithmum maritimum* appears to be a selfing species because its genetic structure is similar to that of most selfing species (Latron et al., 2018). The selfing is a breeding system that assures the reproduction and establishment of a sexual population from even a single colonizer (Crawford et al., 2015), which is advantageous when colonizing volcanic islands. Moreover, selfing species seem to have consistently smaller genome sizes through the reduction in transposable element numbers under the deleterious recessive model of transposable element numbers (Albach & Greilhuber, 2004). This model states that the transposable element number are reduced in selfers due to the greater homozygosity in selfing species, which increase the strength of selection against deleterious insertion that cannot be hidden by recessivity (Albach & Greilhuber, 2004; Morgan, 2001). Therefore, a geographic element related to island isolation and a model of IBD (isolation by distance) seems to be important in shaping the genome size, however, the maintenance through time of such a characteristic may be related to a lack of gene flow (Franks, 2010).

Although further studies are needed to improve our understanding of the mechanisms underlying genome size evolution, our findings seem to point out that smaller genome sizes are correlated with insular environments.

### 4.2 Climatic variables and genome size variation

Here, we present a comparative study of genome size among the mainland populations incorporating environmental variables. The relationships between genome size and both bioclimatic and geographic variables have been widely studied among various groups of plants (Bottini et al., 2000; Brilhante et al., 2021; Díez et al., 2013; Suda et al., 2003, 2005). Hitherto, during the various studies carried out, no universal consistency was reached between them. In our study, relationships were estimated, for the first time, between the genome size of *C. maritimum* and the 22 predictor variables (19 climatic and 3 geographic). It should be noted that geographic variables can be a proxy or involve a complex of several climatic variables that are correlated to them (De Frenne et al., 2013). For mainland populations, climate and geographic position could partly explain the genome size differences. Regarding the different biogeographic regions of Portugal Mainland (Costa et al., 1998), a gradient from north to south can be observed, in fact the median largest genome sizes seem to correspond to the population in the Northernmost sector, the Galician Portuguese Sector, and the smallest median values in the population of the Southernmost sector, the Algarvese‐Monchiquense Sector. A sector is defined by a floristic progression and eventually endemic species, such characteristic is the result of the physical environment (shaped by geology, bioclimatology, and human activities), and therefore, the abovementioned pattern is the result of abiotic factors such as the climate.

Larger genome sizes were found not only in populations located at sites with high temperature variation, high temperature in the driest/warmest part of the year, low temperature in the coldest/wettest part of the year but also in places with low temperatures, high precipitation, and low precipitation seasonality. In other words, genome size tended to increase from south to north. Even in other life forms such as prokaryotes, it was already seen that a greater variability of the environment resulted in genomes with a larger number of genes (Bentkowski et al., 2015). However, it is important to note that these observations should be taken into consideration when extrapolating to other taxa, since negative (e.g., Bottini et al., 2000; Díez et al., 2013) and positive (e.g., Basak et al., 2019; Chrtek Jr. et al., 2009) correlations have been observed for longitude and latitude. The determination of nuclear DNA by flow cytometry can interfered by compounds on plants (Noirot et al., 2000; Price et al., 2000). *C. maritimum* is rich in several compounds such as tanins and flavonoids (Atia et al., 2011), such compounds can interfere with the staining and light scatter properties of the propidium iodide fluorochrome (Loureiro et al., 2006; Peluso et al., 2014). Therefore, an explanation for the variation across the continental part can be due to different chemotypes that already have been observed across the Portuguese coast (Pateira et al., 1999). This artefactual variation, induced by the environment has been observed in *B. bituminosa* (Walker et al., 2006); nevertheless this study also observed true intraspecific related to geographic isolation (Insular vs. Continental populations).

The 114 sampled populations showed a considerably variable pattern of 2C‐values. This variability seems to be shaped by both geographical and bioclimatic variables; that is, some environmental factors such as the characteristics of the growing season might be responsible for that pattern. Nevertheless, the observed pattern between genome size and geographic location was not continuous along the Portuguese coast. A possible reason for this might be efficient seed dispersal by hydrochory (Favre‐Bac et al., 2016), as suggested by Latron et al. (2018, 2020), who observed a lack of spatial trends in the genetic diversity of *C. maritimum*. These genomic variations may be the result of drift or of selection. However, in the case of continental populations, the latter appears to be the main evolutionary force in action, while in the case of the more distinct insular populations, drift, more specifically, founder effect may have played an important role in the mechanisms linked to genome size alteration (Blommaert, 2020).

In conclusions, further studies are needed to improve our understanding of the mechanisms underlying genome size evolution.

## AUTHOR CONTRIBUTIONS

**Guilherme Roxo:** Conceptualization (equal); data curation (equal); formal analysis (equal); investigation (equal); methodology (equal); validation (equal). **Miguel Brilhante:** Formal analysis (equal); methodology (equal); writing – review and editing (equal). **Mónica Maria Tavares Moura:** Investigation (equal); methodology (equal); resources (equal); writing – review and editing (equal). **Miguel Menezes de Sequeira:** Investigation (equal); methodology (equal); resources (equal); writing – review and editing (equal). **Luis Filipe Dias e Silva:** Data curation (equal); formal analysis (equal); methodology (equal); writing – review and editing (equal). **José Carlos Costa:** Investigation (equal); resources (equal); writing – review and editing (equal). **Raquel Vasconcelos:** Investigation (equal); supervision (equal); writing – review and editing (equal). **Pedro Talhinhas:** Formal analysis (equal); investigation (equal); methodology (equal); writing – review and editing (equal). **Maria M. Romeiras:** Conceptualization (equal); funding acquisition (equal); investigation (equal); project administration (equal); resources (equal); supervision (equal); validation (equal); writing – review and editing (equal).

## FUNDING INFORMATION

This research was funded by Fundação para a Ciência e a Tecnologia (FCT) and Aga Khan Development Network (AKDN) under the project CVAgrobiodiversity/333111699. The study was co‐financed by FEDER (85%) and regional funds (15%), through the Azores 2020 Operational Programme, in the context of project eAZFlora (ACORES‐01‐0145‐FEDER‐000007). RV contract was supported by national funds under the scope of “Norma transitória” (DL57/2016/CP1440/CT0002) trough FCT.

## CONFLICT OF INTEREST STATEMENT

The authors declare that they have no conflict of interest.

## Supporting information


**Appendix S1.** Supporting Information

## Data Availability

Genome size (GS) dataset for the 114 populations of *Crithmum maririmum* will be deposited in Zenodo repository once the paper is accepted however a DOI has already been generated: https://doi.org/10.5281/zenodo.7104781.
