# Supplementary material for: Genome size variation within Crithmum maritimum: Clues on the colonization of insular environments
Source: Ecol Evol. 2023 Apr 19;13(4):e10009. doi: 10.1002/ece3.10009 (PMC10116024; doi:10.1002/ece3.10009)
Supplement: Supplementary file 1 — Appendix S1. Supporting Information [file ECE3-13-e10009-s001.docx]

**Supplementary Material Appendix 1**

Genome size variation within *Crithmum maritimum*: clues on the colonization of insular environments

**Guilherme Roxo^1,2,3^, Miguel Brilhante^1^, Mónica Moura^2,3^, Miguel Menezes de Sequeira^4^, Luís Silva^2,3^, José Carlos Costa^1^, Raquel Vasconcelos^3^, Pedro Talhinhas^1^, Maria M. Romeiras^1,5^**

^1^ Linking Landscape, Environment, Agriculture and Food (LEAF), Associated Laboratory TERRA, Instituto Superior de Agronomia (ISA), Universidade de Lisboa, Tapada da Ajuda, 1349-017 Lisboa, Portugal

^2^ CIBIO-Azores, Departamento de Biologia, Universidade dos Açores, Rua Mãe de Deus 58, Apartado 1422, 9501-801 Ponta Delgada, Portugal

^3^ BIOPOLIS Program in Genomics, Biodiversity and Land Planning, CIBIO Centro de Investigação em Biodiversidade e Recursos Genéticos, Campus de Vairão, 4485-661 Vairão, Portugal

^4^ Madeira Botanical Group, Faculty of Life Sciences, University of Madeira, 9020-105 Funchal, Portugal

^5^ Centre for Ecology, Evolution and Environmental Changes (cE3c) & CHANGE - Global Change and Sustainability Institute, Faculdade de Ciências, Universidade de Lisboa, Campo Grande, 1749-016 Lisboa, Portugal

**Contents**

**Table S1.** Details on the sampling locations of the *Crithmum maritimum* populations.

**Table S2.** Description of the environmental variables used in this study.

**Table S3.** Cytogenomic information of the sampled *Crithmum maritimum* populations.

**Figure S1.** Selection of the number of principal components to be retained in a PCA applied to the 19 bioclimatic

| Table S1. Details on the sampling locations of the *Crithmum maritimum* populations. Location: Az, Azores, Ma, Madeira, Lu, mainland, Biogeographic regions: Alt Eur Pro, Atlantic European Province; Mad Pro, Madeirese Province; Alg-Mon Sec, Algarvese-Monchiquense Sector; Rib-Sad Sec, Ribataganian-Sadese Sector; Port Div Sec, Portuguese Divisorian Sector; Gal Por Sec, Galician Portuguese Sector. | | | | |
| --- | --- | --- | --- | --- |
| Location | **‘Distrito’/ Island** | **Municipality** | **Locality** | Biogeographic Region |
| Az | Corvo | Corvo | Vila do Corvo | Alt Eur Pro |
| Az | Faial | Horta | Pedro Miguel | Alt Eur Pro |
| Az | Faial | Horta | Ponta Furada | Alt Eur Pro |
| Az | Pico | Lajes do Pico | Companhia de Cima | Alt Eur Pro |
| Az | Pico | Lajes do Pico | Poça das Mujas | Alt Eur Pro |
| Az | Pico | Madalena do Pico | Criação Velha | Alt Eur Pro |
| Az | Pico | Madalena do Pico | Guindaste | Alt Eur Pro |
| Az | Pico | Madalena do Pico | Porto do Calhau | Alt Eur Pro |
| Az | Pico | Madalena do Pico | São Mateus | Alt Eur Pro |
| Az | Pico | São Roque do Pico | Lajido | Alt Eur Pro |
| Az | Pico | São Roque do Pico | Ponta do Boqueirão | Alt Eur Pro |
| Az | Santa Maria | Vila do Porto | Anjos | Alt Eur Pro |
| Az | Santa Maria | Vila do Porto | Furna de Santana | Alt Eur Pro |
| Az | São Jorge | Calheta | Calheta | Alt Eur Pro |
| Az | São Miguel | Lagoa | Caloura | Alt Eur Pro |
| Ma | Deserta Grande | Santa Cruz | Deserta Grande | Mad Pro |
| Ma | Madeira | Calheta | Paul do Mar | Mad Pro |
| Ma | Madeira | Funchal | Lido | Mad Pro |
| Ma | Madeira | Funchal | Praia Formosa | Mad Pro |
| Ma | Madeira | Machico | Machico | Mad Pro |
| Ma | Madeira | Machico | Ponta de São Lourenço | Mad Pro |
| Ma | Madeira | Machico | Porto da Cruz | Mad Pro |
| Ma | Madeira | Ponta do Sol | Ponta do Sol | Mad Pro |
| Ma | Madeira | Porto Moniz | Fajã das Achadas da Cruz | Mad Pro |
| Ma | Madeira | Porto Moniz | Parque de Campismo do Porto Moniz | Mad Pro |
| Ma | Madeira | Porto Moniz | Piscinas Naturais do Seixal | Mad Pro |
| Ma | Madeira | Porto Moniz | Piscinas Naturais velhas do Porto Moniz | Mad Pro |
| Ma | Madeira | Porto Moniz | Praia do Seixal | Mad Pro |
| Ma | Madeira | Santana | Faial | Mad Pro |
| Ma | Madeira | Santana | Rocha de Baixo | Mad Pro |
| Ma | Madeira | Santana | Ruínas de São Jorge | Mad Pro |
| Ma | Madeira | São Vicente | Estrada Antiga Ponta Delgada - São Vicente | Mad Pro |
| Ma | Madeira | São Vicente | Piscinas da Ponta Delgada | Mad Pro |
| Ma | Madeira | São Vicente | São Vicente | Mad Pro |
| Ma | Porto Santo | Porto Santo | Praia da Lagoa | Mad Pro |
| Lu | Faro | Lagoa | Praia dos Caneiros | Alg-Mon Sec |
| Lu | Faro | Portimão | Prainha | Alg-Mon Sec |
| Lu | Faro | Portimão | Praia dos Três Irmãos | Alg-Mon Sec |
| Lu | Faro | Vila do Bispo | Praia do Beliche | Alg-Mon Sec |
| Lu | Faro | Vila do Bispo | Cabo de São Vicente | Alg-Mon Sec |
| Lu | Beja | Odemira | Porto das Barcas | Rib-Sad Sec |
| Lu | Setúbal | Grândola | Praia do Carvalhal | Rib-Sad Sec |
| Lu | Setúbal | Alcácer do Sal | Praia da Comporta | Rib-Sad Sec |
| Lu | Setúbal | Alcochete | Praia do Samouco | Rib-Sad Sec |
| Lu | Lisboa | Lisboa | Belém | Port Div Sec |
| Lu | Lisboa | Oeiras | Paço de Arcos | Port Div Sec |
| Lu | Lisboa | Oeiras | Praia da Torre | Port Div Sec |
| Lu | Lisboa | Cascais | Praia de Carcavelos | Port Div Sec |
| Lu | Lisboa | Cascais | Praia da Parede | Port Div Sec |
| Lu | Lisboa | Cascais | Praia das Avencas | Port Div Sec |
| Lu | Lisboa | Cascais | São João do Estoril | Port Div Sec |
| Lu | Lisboa | Cascais | Boca do Inferno | Port Div Sec |
| Lu | Lisboa | Cascais | Guincho | Port Div Sec |
| Lu | Lisboa | Sintra | Praia das Maçãs | Port Div Sec |
| Lu | Lisboa | Sintra | Azenhas do Mar | Port Div Sec |
| Lu | Leiria | Peniche | Fortaleza de Peniche | Port Div Sec |
| Lu | Leiria | Peniche | Farol do Cabo Carvoeiro | Port Div Sec |
| Lu | Leiria | Peniche | Papôa | Port Div Sec |
| Lu | Leiria | Peniche | Forte de São João Batista (Berlenga Grande) | Port Div Sec |
| Lu | Leiria | Peniche | Melreu (Berlenga Grande) | Port Div Sec |
| Lu | Leiria | Caldas da Rainha | Foz do Arelho | Port Div Sec |
| Lu | Leiria | Alcobaça | São Martinho do Porto | Port Div Sec |
| Lu | Leiria | Nazaré | Praia da Nazaré | Port Div Sec |
| Lu | Leiria | Nazaré | Praia do Norte | Port Div Sec |
| Lu | Leiria | Alcobaça | Praia de Paredes de Vitória | Port Div Sec |
| Lu | Leiria | Alcobaça | Praia da Polvoleira | Port Div Sec |
| Lu | Leiria | Marinha Grande | São Pedro de Moel | Port Div Sec |
| Lu | Leiria | Marinha Grande | Praia Vieira de Leiria | Port Div Sec |
| Lu | Leiria | Leiria | Praia do Pedrogão | Port Div Sec |
| Lu | Coimbra | Figueira da Foz | Praia do Cabedelo | Port Div Sec |
| Lu | Coimbra | Figueira da Foz | Praia da Claridade | Port Div Sec |
| Lu | Coimbra | Figueira da Foz | Praia Laje do Costado | Port Div Sec |
| Lu | Porto | Vila Nova de Gaia | Praia de Valadares Norte | Gal Por Sec |
| Lu | Porto | Vila Nova de Gaia | Praia de Salgueiros | Gal Por Sec |
| Lu | Porto | Vila Nova de Gaia | Pedra do Cão | Gal Por Sec |
| Lu | Porto | Porto | Forte de São João | Gal Por Sec |
| Lu | Porto | Porto | Forte de São João Baptista | Gal Por Sec |
| Lu | Porto | Porto | Foz do Rio Douro | Gal Por Sec |
| Lu | Porto | Porto | Forte de São Francisco Xavier | Gal Por Sec |
| Lu | Porto | Matosinhos | Praia da Senhora da Boa Nova | Gal Por Sec |
| Lu | Porto | Matosinhos | Praia Cabo do Mundo | Gal Por Sec |
| Lu | Porto | Matosinhos | Praia da Memória | Gal Por Sec |
| Lu | Porto | Matosinhos | Praia da Agudela | Gal Por Sec |
| Lu | Porto | Matosinhos | Praia do Funtão | Gal Por Sec |
| Lu | Porto | Matosinhos | Praia dos Barcos | Gal Por Sec |
| Lu | Porto | Matosinhos | Praia Angeiras Norte | Gal Por Sec |
| Lu | Porto | Vila do Conde | Praia Castro de São Paio | Gal Por Sec |
| Lu | Porto | Vila do Conde | Praia de Vila Chã | Gal Por Sec |
| Lu | Porto | Vila do Conde | Praia da Laderça | Gal Por Sec |
| Lu | Porto | Vila do Conde | Praia de Mindelo | Gal Por Sec |
| Lu | Porto | Vila do Conde | Praia da Árvore | Gal Por Sec |
| Lu | Porto | Vila do Conde | Foz do Rio Ave | Gal Por Sec |
| Lu | Porto | Vila do Conde | Praia Azul Norte | Gal Por Sec |
| Lu | Porto | Póvoa do Varzim | Fortaleza da Póvoa de Varzim | Gal Por Sec |
| Lu | Porto | Póvoa do Varzim | Praia do Fragosinho | Gal Por Sec |
| Lu | Porto | Póvoa do Varzim | Cabo de Santo André | Gal Por Sec |
| Lu | Porto | Póvoa do Varzim | Praia de Aver-o-Mar | Gal Por Sec |
| Lu | Viana do Castelo | Viana do Castelo | Praia do Coral | Gal Por Sec |
| Lu | Viana do Castelo | Viana do Castelo | Forte da Areosa | Gal Por Sec |
| Lu | Viana do Castelo | Viana do Castelo | Moinho do Vento do Canto Marinho | Gal Por Sec |
| Lu | Viana do Castelo | Viana do Castelo | Praia do Lumiar | Gal Por Sec |
| Lu | Viana do Castelo | Viana do Castelo | Praia de Fornelos e Promontório de Montedor | Gal Por Sec |
| Lu | Viana do Castelo | Viana do Castelo | Praia do Paçô | Gal Por Sec |
| Lu | Viana do Castelo | Viana do Castelo | Praia da Ínsua | Gal Por Sec |
| Lu | Viana do Castelo | Caminha | Praia Forte do Cão | Gal Por Sec |
| Lu | Viana do Castelo | Caminha | Praia da Âncora | Gal Por Sec |
| Lu | Viana do Castelo | Caminha | Vila Praia de Âncora | Gal Por Sec |
| Lu | Viana do Castelo | Caminha | Forte da Lagarteira | Gal Por Sec |
| Lu | Viana do Castelo | Caminha | Capela de Santo Isidoro | Gal Por Sec |
| Lu | Viana do Castelo | Caminha | Praia de Moledo | Gal Por Sec |
| Lu | Viana Do Castelo | Caminha | Foz do Minho | Gal Por Sec |
| Lu | Viana do Castelo | Caminha | Praia do Camarido | Gal Por Sec |
| Lu | Viana do Castelo | Caminha | Caminha | Gal Por Sec |
| Lu | Viana do Castelo | Caminha | Marina de Seixas | Gal Por Sec |

| **Table S2.** Description of the environmental variables used in this study, their code and units. The first 19 were obtained from CHELSA (Karger *et al.*, 2017), their codes and units and the last three from GPS data. | | |
| --- | --- | --- |
| **Description** | **Code** | **Units** |
| Annual Mean Temperature | BIO01 | °C*10 |
| Mean Diurnal Range^1^ | BIO02 | °C |
| Isothermality^2^ | BIO03 | dimensionless |
| Temperature Seasonality^3^ | BIO04 | °C*100 |
| Maximum Temperature of Warmest Month | BIO05 | °C*10 |
| Minimum Temperature of Coldest Month | BIO06 | °C*10 |
| Temperature Annual Range^4^ | BIO07 | °C*10 |
| Mean Temperature of Wettest Quarter | BIO08 | °C*10 |
| Mean Temperature of Driest Quarter | BIO09 | °C*10 |
| Mean Temperature of Warmest Quarter | BIO10 | °C*10 |
| Mean Temperature of Coldest Quarter | BIO11 | °C*10 |
| Annual Precipitation | BIO12 | mm/year |
| Precipitation of Wettest Month | BIO13 | mm/month |
| Precipitation of Driest Month | BIO14 | mm/month |
| Precipitation Seasonality^5^ | BIO15 | coefficient of variation |
| Precipitation of Wettest Quarter | BIO16 | mm/quarter |
| Precipitation of Driest Quarter | BIO17 | mm/quarter |
| Precipitation of Warmest Quarter | BIO18 | mm/quarter |
| Precipitation of Coldest Quarter | BIO19 | mm/quarter |
| Latitude | Lat | Decimal degrees |
| Longitude | Long | Decimal degrees |
| Altitude | Alt | m |

^1^Mean of monthly (Maximum temperature ‐ Minimum temperature)

^2^ (BIO02/BIO07) (*100)

^3^ Standard deviation of monthly temperature averages

^4^ (BIO05 – BIO06)

^5^ Standard deviation of monthly precipitation averages

| Table S3. Mean 2C-values (in picograms, pg), standard deviation (SD), and coefficient of variation (CV, in percentage, %) of *Crithmum maritimum* populations. Acronyms between brackets stand for Azores archipelago (Az), Madeira archipelago (Ma) and Portugal Mainland (Lu). | | | |
| --- | --- | --- | --- |
| Location | **Mean** | **SD** | **Sample CV (%)** |
| (Ma) Madeira (Fajã das Achadas da cruz) | 4.074 | 0.079 | 3.076 |
| (Az) Pico (Criação Velha) | 4.098 | 0.052 | 3.434 |
| (Az) Pico (Guindaste) | 4.113 | 0.075 | 3.053 |
| (AZ) Santa Maria (Anjos) | 4.167 | 0.039 | 2.739 |
| (Az) Pico (Porto do Calhau) | 4.171 | 0.059 | 3.069 |
| (AZ) Faial (Pedro Miguel) | 4.175 | 0.100 | 6.337 |
| (Az) Pico (Poça das Mujas) | 4.191 | 0.036 | 3.252 |
| (Az) Pico (Ponta do Boqueirão) | 4.217 | 0.071 | 3.369 |
| (Ma) Desertas (Deserta Grande) | 4.233 | 0.063 | 0.840 |
| (Az) São Miguel (Caloura) | 4.235 | 0.044 | 2.733 |
| (Az) Pico (Lajido) | 4.240 | 0.052 | 3.238 |
| (Az) Corvo | 4.256 | 0.041 | 3.627 |
| (Az) São Jorge (Calheta) | 4.258 | 0.090 | 2.370 |
| (Az) Pico (Companhia de Cima) | 4.265 | 0.042 | 1.345 |
| (Ma) Madeira (Ponta do Sol) | 4.283 | 0.021 | 2.688 |
| (Ma) Madeira (Praia do Seixal) | 4.291 | 0.079 | 2.545 |
| (Az) Santa Maria (Furna de Santana) | 4.315 | 0.025 | 2.270 |
| (Ma) Madeira (Ruínas de S. Jorge) | 4.321 | 0.081 | 2.725 |
| (Ma) Madeira (Parque de Campismo do Porto Moniz) | 4.323 | 0.070 | 2.239 |
| (Az) Faial (Ponta Furada) | 4.329 | 0.098 | 6.928 |
| (Ma) Madeira (Praia Formosa) | 4.329 | 0.090 | 3.008 |
| (Ma) Madeira (Piscinas Naturais do Seixal) | 4.331 | 0.075 | 2.397 |
| (Ma) Madeira (Porto da Cruz) | 4.335 | 0.074 | 2.761 |
| (Ma) Madeira (Lido) | 4.352 | 0.069 | 2.851 |
| (Ma) Madeira (Ponta de São Lourenço) | 4.353 | 0.043 | 2.601 |
| (Ma) Madeira (Rocha de baixo) | 4.354 | 0.063 | 2.649 |
| (Az) Pico (São Mateus) | 4.362 | 0.448 | 2.609 |
| (Ma) Madeira (Piscinas da Ponta Delgada) | 4.363 | 0.089 | 2.444 |
| (Ma) Madeira (Machico) | 4.377 | 0.077 | 3.073 |
| (Ma) Porto Santo (Praia da Lagoa) | 4.378 | 0.109 | 2.535 |
| (Ma) Madeira (Piscinas Naturais velhas do Porto Moniz) | 4.393 | 0.064 | 2.281 |
| (Ma) Madeira (Faial) | 4.407 | 0.081 | 2.519 |
| (Ma) Madeira (Estrada antiga de São Vicente) | 4.434 | 0.125 | 2.349 |
| (Ma) Madeira (São Vicente) | 4.470 | 0.082 | 2.799 |
| (Lu) Lisboa (Praia de Carcavelos) | 4.472 | 0.077 | 2.766 |
| (Lu) Leiria (Foz do Arelho) | 4.499 | 0.120 | 2.779 |
| (Ma) Madeira (Paul do Mar) | 4.511 | 0.085 | 2.830 |
| (Lu) Lisboa (Praia da Parede) | 4.525 | 0.075 | 3.268 |
| (Lu) Lisboa (São João do Estoril) | 4.575 | 0.050 | 2.746 |
| (Lu) Beja (Praia do Carvalhal) | 4.580 | 0.079 | 4.830 |
| (Lu) Lisboa (Praia das Maçãs) | 4.582 | 0.056 | 2.772 |
| (Lu) Lisboa (Azenhas do Mar) | 4.618 | 0.162 | 2.618 |
| (Lu) Faro (Praia dos Carneiros) | 4.635 | 0.051 | 2.601 |
| (Lu) Lisboa (Praia da Torre) | 4.652 | 0.070 | 3.293 |
| (Lu) Lisboa (Praia das Avencas) | 4.658 | 0.103 | 2.548 |
| (Lu) Lisboa (Boca do Inferno) | 4.679 | 0.047 | 3.328 |
| (Lu) Leiria (Praia do Pedrogão) | 4.729 | 0.112 | 2.409 |
| (Lu) Faro (Praia do Beliche) | 4.729 | 0.044 | 2.839 |
| (Lu) Viana do Castelo (Caminha) | 4.738 | 0.228 | 2.295 |
| (Lu) Faro (Prainha) | 4.746 | 0.093 | 2.626 |
| (Lu) Leiria (Praia da Nazaré) | 4.764 | 0.066 | 2.629 |
| (Lu) Beja (Porto das Barcas) | 4.764 | 0.045 | 4.667 |
| (Lu) Leiria (Praia do Norte) | 4.788 | 0.098 | 2.306 |
| (Lu) Coimbra (Praia da Claridade) | 4.801 | 0.057 | 2.684 |
| (Lu) Porto (Pedra do Cão) | 4.811 | 0.087 | 2.133 |
| (Lu) Viana do Castelo (Praia de Fornelos e Promontório de Montedor) | 4.817 | 0.060 | 2.281 |
| (Lu) Viana do Castelo (Praia da Ínsua) | 4.820 | 0.065 | 1.759 |
| (Lu) Viana do Castelo (Praia do Camarido) | 4.828 | 0.075 | 2.010 |
| (Lu) Lisboa (Guincho) | 4.834 | 0.046 | 1.357 |
| (Lu) Viana do Castelo (Praia do Paçô) | 4.838 | 0.057 | 2.096 |
| (Lu) Porto (Praia de Valadares Norte) | 4.841 | 0.105 | 2.311 |
| (Lu) Porto (Forte de São Francisco Xavier) | 4.844 | 0.074 | 2.115 |
| (Lu) Porto (Foz do Rio Ave) | 4.855 | 0.133 | 2.230 |
| (Lu) Leiria (Farol do Cabo Carvoeiro) | 4.855 | 0.098 | 3.019 |
| (lu) Porto (Praia de Aver-o-Mar) | 4.857 | 0.072 | 1.670 |
| (Lu) Viana do Castelo (Forte da Areosa) | 4.857 | 0.066 | 1.987 |
| (Lu) Viana do Castelo (Capela de Santo Isidoro) | 4.864 | 0.083 | 2.375 |
| (Lu) Lisboa (Belém) | 4.865 | 0.038 | 2.557 |
| (Lu) Faro (Praia dos Três Irmãos) | 4.867 | 0.023 | 2.230 |
| (Lu) Viana do Castelo (Praia da Âncora) | 4.867 | 0.063 | 2.277 |
| (Lu) Leiria (Praia Vieira de Leiria) | 4.870 | 0.120 | 2.224 |
| (Lu) Porto (Cabo de Santo André) | 4.872 | 0.055 | 1.962 |
| (Lu) Viana do Castelo (Praia do Lumiar) | 4.875 | 0.087 | 2.343 |
| (Lu) Porto (Praia de Salgueiros) | 4.878 | 0.073 | 2.171 |
| (Lu) Faro (Cabo de São Vicente) | 4.880 | 0.228 | 2.470 |
| (Lu) Leiria (Praia do Samouco) | 4.882 | 0.091 | 2.722 |
| (Lu) Leiria (Praia da Polvoleira) | 4.884 | 0.128 | 2.033 |
| (Lu) Leiria (São Martinho do Porto) | 4.895 | 0.079 | 2.001 |
| (Lu) Leiria (Papôa, Peniche) | 4.897 | 0.128 | 2.397 |
| (Lu) Porto (Praia Angeiras Norte) | 4.899 | 0.143 | 2.207 |
| (Lu) Coimbra (Praia Lajde do Costado) | 4.899 | 0.129 | 2.294 |
| (Lu) Coimbra (Praia do Cabedelo) | 4.901 | 0.067 | 2.511 |
| (Lu) Porto (Fortaleza da Póvoa de Varzim) | 4.904 | 0.079 | 2.431 |
| (Lu) Viana Do Castelo (Foz do Minho) | 4.905 | 0.055 | 2.237 |
| (Lu) Porto (Forte de São João Baptista) | 4.908 | 0.141 | 1.987 |
| (Lu) Leiria (Praia de Paredes de Vitória) | 4.910 | 0.065 | 2.388 |
| (Be) Berlenga Grande (Forte de São João Baptista) | 4.912 | 0.095 | 2.241 |
| (Lu) Viana do Castelo (Praia de Moledo) | 4.915 | 0.075 | 2.206 |
| (Lu) Lisboa (Paço de Arcos) | 4.921 | 0.045 | 2.477 |
| (Lu) Porto (Praia do Funtão) | 4.927 | 0.127 | 2.094 |
| (Lu) Porto (Praia Azul Norte) | 4.928 | 0.068 | 2.119 |
| (Lu) Porto (Praia Castro de São Paio) | 4.933 | 0.094 | 2.298 |
| (Lu) Porto (Foz do Rio Douro) | 4.934 | 0.063 | 1.951 |
| (Lu) Viana do Castelo (Marina de Seixas) | 4.935 | 0.042 | 1.919 |
| (Lu) Viana do Castelo (Praia Forte do Cão) | 4.943 | 0.065 | 2.123 |
| (Lu) Viana do Castelo (Moinho do Vento do Canto Marinho) | 4.943 | 0.084 | 2.114 |
| (Lu) Leiria (Fortaleza de Peniche) | 4.945 | 0.048 | 2.444 |
| (Lu) Setúbal (Praia da Comporta) | 4.946 | 0.099 | 2.149 |
| (Lu) Porto (Praia Cabo do Mundo) | 4.946 | 0.065 | 2.052 |
| (Lu) Porto (Forte de São João) | 4.963 | 0.081 | 2.303 |
| (Lu) Porto (Praia da Agudela) | 4.966 | 0.103 | 1.951 |
| (Be) Berlenga Grande (Melreu) | 4.972 | 0.037 | 2.001 |
| (Lu) Porto (Praia de Mindelo) | 4.979 | 0.086 | 2.059 |
| (Lu) Porto (Praia da Laderça) | 4.999 | 0.097 | 2.100 |
| (Lu) Porto (Praia da Senhora da Boa Nova) | 5.000 | 0.049 | 2.041 |
| (Lu) Porto (Praia do Fragosinho) | 5.002 | 0.057 | 2.201 |
| (Lu) Porto (Praia da Árvore) | 5.005 | 0.054 | 2.157 |
| (Lu) Porto (Praia da Memória) | 5.008 | 0.076 | 2.410 |
| (Lu) Porto (Praia de Vila Chã) | 5.017 | 0.095 | 2.404 |
| (Lu) Viana do Castelo (Praia do Coral) | 5.022 | 0.070 | 2.475 |
| (Lu) Viana do Castelo (Vila Praia de Âncora) | 5.023 | 0.073 | 1.949 |
| (Lu) Leiria (São Pedro de Moel) | 5.034 | 0.156 | 2.424 |
| (Lu) Viana do Castelo (Forte da Lagarteira) | 5.041 | 0.081 | 2.107 |
| (Lu) Porto (Praia dos Barcos) | 5.047 | 0.145 | 2.181 |
| Average | 4.710 | 0.294 | 2.445 |

**Mainland**

**All data**

**C**

**A**

**D**

**B**

**Figure S1**. Selection of the number of principal components to be retained in a PCA applied to the 19 bioclimatic variables, according to the Kaiser-Guttman criterion (**A, C**) and the broken stick model (**B, D**) for the full data including islands and mainland, and for data including mainland only, respectively. In (**A, B**) three components were retained which explain 51.2%, 26.2% and 13.8% and in (**C, D**) two components were retained which explain 60.7% and 32.7% of the variation in the data, respectively.
